# A Collection of Surgical Memoirs

**Published:** 1822-12-01

**Authors:** 


*Recueil de Memoires de Chirurgie, par le* Baron D. J. Larrey, Chirurgien en chef de l'Hopital de la Garde Royle, &c. &c. &c. &c. *Paris,* 1821, pp. 319.

It has been frequently and justly observed, that the laws of social benevolence require that every man should endeavour to assist others by his experience.

Notwithstanding the truth of this proposition is generally, nay, perhaps, invariably admitted, it is not uncommon for those, whom nature has blest with talent, and fortune favoured with numerous opportunities of benefiting mankind by the promulgation of their practical experience, to sink into a state of listless apathy during their more leisure hours, without considering it necessary to dedicate a single moment to the fulfilment of what may justly be deemed an imperious moral duty—namely to the improvement of the profession to which they belong. To behold others with frigid indifference, struggling with difficulties which we could teach them to overcome—to witness failure when we could impart the means of success, is certainly however not the characteristic of the medical profession. Baron Larrey is a prominent example of unwearied industry, and professional zeal. He no sooner obtains information, than he hastens, in the true spirit of philanthropy, to impart it to his brethren. Since the publication of the last volume of his campaigns, he has been sedulously occupied in collecting additional evidence, upon some subjects which he merely glanced at in that important work. The first memoir in the pesent volume is upon moxa ; its mode of application is pointed out, and various cases are detailed of those diseases, in which its use has been found advantageous. We highly approve of the cautious scrutiny to which the merits of this application have been subjected. It is by far too painful, and too unpleasant a remedy, to be had recourse to upon light authority, or without sufficient proofs of its efficacy. Foreign surgeons, have at length, the Baron observes, recognised the advantages to be derived from moxa, and have adopted the use of it as the best mode of treating several chronic affections which have hitherto been considered incurable, such as diseases of the spine—of the hip joint—phthisis pulmonis—scirrhous of the pylorus, &c. The inhabitants of Asia and Africa have the highest confidence in the application of this remedy. They use it, not only for the purpose of removing diseases which are incurable by other means, but also to *pre-*

*vent* their occurrence. It is not improbable that it has fallen into disrepute in Europe, in consequence of having been improperly and carelessly applied. We refer our readers to the "*Dict. des Sciences Med.*" Art. "Moxibustion," for some interesting details descriptive of the various ways, in which it is applied in different countries.

The cone or cylinder of moxa, is composed of cotton rolled upon a small quantity of fine linen, stitched at the edge. It should be about an inch long and of a proportionate thickness. Its size, however, may be varied according to circumstances. A moxa frame, of which an engraving is given, is employed to apply it to the precise spot required. The metallic ring of the moxa frame is insulated from the skin by three small ebony balls. After having lighted the extremity of the cone, combustion is kept up by means of a blow-pipe. Rapid combustion is not desirable.

The spot upon which the moxa is to be applied is first to be marked with ink, the surrounding parts being protected from injury by being covered with pieces of damp linen. To prevent the deep seated inflammation and suppuration which would result, liquor anmoniæ is to be immediately applied, when the combustion of the moxa has been effected. According to some authors moxa may be applied indiscriminately to all parts of the body. Baron Larrey excepts all that part of the cranium which is covered only with the integuments and pericranium. Two cases are related by Dehaen in proof of the danger, of applying a cautery to this part. Moxa should not be applied to the eyelids, nose, or ears. It is also necessary in the opinion of the Baron, to avoid the course of the larynx, and trachea, the sternum, the glandular parts of the breasts, the linea alba of the abdomen, and the organs of generation.

It may, however, be applied to the perineum, near the origin of the urethra, for chronic scirrhous affections particularly of the prostate gland. We should also abstain from the application of any kind of cautery over the course of superficial tendons, or near the articulations, where we should incur the danger of injuring the capsules of the joint. The properties of moxa differ from those of the metallic cautery, the effects of which are confined to the burned part, which is more or less disorganized according to the size and thickness of the cautery, and the force with which it is applied.

Moxa is considered much less painful than the metallic cautery, besides which it is said to communicate to the parts, "avec une masse relative de calorique, un principe volatil tres-actif, que fournissent les substances cotonneuses, lorsqu'elles sont en combustion."

This we think is rather a fanciful mode of explaining the peculiar advantages of moxa. This "*principe volatil*" furnished by cottony substances whilst burning, must be immensely abundant and active, to be productive of any particular benefit from the combustion of so small a quantity of cotton.

If it is required to obtain merely a superficial effect, the moxa is allowed to burn without making use of the blowpipe. Baron Percy, our author's colleague, uses it in this manner. The sensation which first arises from the application of moxa, is, we are told, "plutot une sensation agréable que de la douleur." That very severe pain quickly follows is not denied; it is borne however with fortitude in most cases, as the patient knows, after the first application, that it is *instantly* relieved by liquor ammoniæ. The number of moxas required will depend upon the nature of the disease. One only, or two at most, should be applied at the same time. Several days should intervene between each application.

The good effects of this remedy are, in many cases, increased by the previous application of cupping glasses to the part, with or without scarification.

After these preliminary observations, the Baron enters upon a brief description of cases in which he considers the moxa required. He commences with diseases of the organs of sensation, and, first—

*Of Vision.* Incipient cataract, or recent paralysis of the optic nerves particularly demand the use of moxa, which should be applied over the course of the nerves most connected with those of the eye, such as the trunk and principal branches of the superior maxillary, and frontal nerves. By the use of moxa, says the Baron, the progress of amaurosis has not only been arrested, but some cases have been completely cured, in which the blindness was complete. An interesting case illustrative of this statement is adduced, and similar instances are related by the author in his "*Memoires Chirurgicales.*" We are advised to *second* the effects of moxa by the appropriate remedies, such as previous cupping, the internal use of calomel, electricity, &c.

We are not inclined to attempt even the Herculean task of reconciling the discrepancy of opinion which we have such frequent cause to lament. We should not, however, feel ourselves justified in withholding from our readers the fact, that our able and assiduous friend Mr. Guthrie, who has extensive practice as an oculist, both public and private, has tried the moxa largely for near six years, and that he has scarcely seen a single instance in which it has been of permanent utility in amaurosis, or any other disease of the eye. Baron Larrey has derived no benefit from moxa, in affections of the olfactory and gustatory nerves. Deafness and loss of speech arising from the sudden application of cold, &c. have been frequently cured by its employment.

"*Paralytic affections of the Muscular System.*" The effects of moxa in paralytic affections, with or without neuralgia, are next considered. Three cases of severe tic douloureux, of long standing, are related. They had resisted all the usual remedies, and are considered to have been cured by moxa repeatedly applied. It should be observed, however, that in two of these cases, the general health was considerably impaired by the continuance of this distressing malady, and that, previous to the use of moxa, the Baron very properly directed his attention to the cure of the constitutional derangement. When we consider the intimate relation that exists between such local affections, and the state of the constitution, we cannot but ascribe a great part of the benefit, to the general treatment so judiciously enforced by our author. The use of moxa "is not indicated in acute neuralgia, arising from spontaneous causes, nor in tetanic affections. It augments the irritation and tetanus. We have employed it to no purpose in the latter disease." An interesting case of disease of the spine is related, accompanied with paralysis, which was cured by thirty-two applications of moxa. In mentioning the previous treatment of this case, the Baron observes in a note "L'essai que j'ai vouler faire de ce remède (la noix vomìque) chez quelques-une de nos paralytiques, a produit le même résultat. Il n'est pas douteux que loin de dissiper la phlegmasies des membranes nerveuses, elle ne l'augmente; j'ai remarqué que ses effets sont constamment pernicieux, et je pense, contre l'opinion de quelques médecins quì préconìsert cependant ce remède, qu'il est un de ceux dont l'usage doit être proscrit en medecine."

Notwithstanding this sweeping reprobation of the nux vomica, we can declare from our own experience, that it may at least be exhibited without danger. The experiments of Drs. Magendie, Delile, Desportes, and Orfila, prove that the nux vomica exercises a peculiar action upon the spinal marrow. Dr. Fouquier, and M. Bricheteau, considered it as a valuable remedy in paralysis; they have administered it in many cases.* Two cases of paralysis of the fore arm from

---

* Vide our Journal for October, 1818.

gun shot wounds, in which the sensibility of the parts was highly increased were cured by moxa. The torments endured by the subjects of these cases, and the failure of every remedy, made them anxious for amputation; fortunately, however, they submitted to the use of moxa, and it proved completely successful. Hemiplegia of the face, Baron Larrey observes, had hitherto been considered incurable by authors. The application of moxa to this part was thought dangerous, and justly so, unless the cones of cotton were used of a smaller size, and suppuration prevented by the application of ammonia.

Several young soldiers of the Imperial Guard, who were attacked with hemiplegia of the face in consequence of bivouacking in damp situations, were cured by moxa. The following interesting case we give in the words of our author.

"Mad. de M. aged 17 years, of a nervous and delicate constitution, had been affected since her infancy, with paralysis of the left side of the face, which took place at the termination of a worm fever. For this, electricity and mineral waters were tried in vain. The deformity was great, and gave to this young woman, who was otherwise handsome, a very disagreeable aspect especially when she smiled. Baron Larrey applied some moxa cones, at first, along the track of the facial nerve as it passes out of the stylo-mastoid foramen—and afterwards in three divergent lines, following the course of the principal branches of the same nerve. The pain was great, but the young patient bore it without uttering a word. The application of liquid volatile alkali immediately removed the pain when the moxa was taken off, and the eschars separated on the 10th to the 13th days, leaving very small red cicatrices which disappeared in time. After the fourth application there was a sensible change for the better; but the amelioration was very gradual, till the ninth repetition of the moxa, when the amendment was rapid, and by the 17th, the patient might be considered as cured, so trifling were the vestiges of the former deformity." 94.

This is an unquestionable proof of the occasional efficacy of moxa. We fear, however, that few patients would submit to so formidable a mode of treatment, excepting indeed, a young lady anxious for the restoration of her beauty. It should be observed, that we are advised to prevent suppuration from the use of moxa in simple paralytic affections, unattended by neuralgia, while it is considered beneficial in painful paralysis. Complete success, says the Baron, results from the use of moxa, in cases of paraplegia depending upon affections of the spinal marrow, "lorsqu'elles ne sont pas tres anciennes, et qu'elles ne sont pas compliquées d'incontinence d'urine, symptome tres facheux." We must refer to the work itself for a very striking case of paraplegia in the person of a general officer, which was successfully treated by moxa. The Baron next enters upon a rapid sketch of the organic diseases in which moxa has been useful, and to our astonishment commences the chapter in these words. "In *all* chronic affections of the head, I have employed this remedy with the greatest success. In idiopathic epilepsy, *dropsy of the ventricles of the brain*, chronic cephalalgia, &c. &c. the moxa, should be applied all round the basis of the cranium, and particularly at the junction of the squamous suture with the lambdoidal." We pass over the cases of head affections, which are related as proofs of the efficacy of moxa. As such, they are to us extremely unsatisfactory; their nature is problematical, and in each of them copious bleedings, blisters, calomel, the long continued application of ice, &c. were had recourse to previous to the use of moxa, which we conceive to have had little claim to the ultimate recovery of the patients.

By some authors the use of moxa is recommended in mental diseases attended with excitement. The Baron disapproves of the remedy in such cases.

*Diseases of the Chest.* Asthma, painful palpitations of the heart, and chronic inflammations of the pleura, are said to have been relieved by moxa. That counter-irritation, excited in the ordinary manner, is productive of much benefit in such cases, is an admitted fact, and we are still somewhat sceptical as to the superior efficacy of moxa.

*Phthisis pulmonalis* is the next disease considered as frequently remediable by moxa. Upon this subject the Baron is brief, as it is his intention to publish a separate treatise upon it, as soon as he has collected sufficient materials. To scrophulous and rheumatic inflammations of the hip joint, &c. &c. the term "phthisis of the bones" is applied, and our author observes, that if attention be paid to the success which has resulted from the use of moxa in such cases, we shall be convinced of its efficacy in pulmonary phthisis, which differs from those affections "only in its seat." For our own parts, we certainly should *not* confidently rely upon a particular mode of treatment in phthisis pulmonalis, *because* it had been successfully employed in diseases of the vertebræ, or hip joint, &c.

That "the same phenomena are occasionally observed in these affections, the same causes recognized, and the same effects produced," is very true, but surely the result of any mode of treatment will be very materially modified, if not rendered totally different, from the different structure, functions, and importance to the animal economy, of the affected parts. The constant action of the lungs, also, presents a formidable impediment to our curative endeavours, which we have not to encounter in the other diseases mentioned by the author. Abstractedly considered, we might not, perhaps, have objected to the application of the term "Phthisis of the bones;" if, however, it is to be applied for the purpose of leading us on to a confession of the identity of vertebral disease, &c. with pulmonary consumption, with the single exception of the difference of situation, and to the inference, that a similar mode of treatment will, in each case, be productive of similar effects, we decidedly disapprove of it, as being likely to lead to very erroneous analogical reasoning, and inappropriate practice. The Baron incidentally remarks, that the superacetate of lead, and more particularly the prussic acid, both of which have been so highly extolled in phthisis pulmonalis, are generally hurtful, or at least useless. In a public institution with which we are acquainted, and in our own more limited private practice, the latter remedy has been given in different stages of phthisis without the least advantage. As it is, of course, desirable to apply the moxa as nearly as possible over the diseased part of the lungs, the stethescope of Laennec is recommended as facilitating the investigation, although, as our author justly observes, the experienced physician will not require its assistance. Percussion and careful pressure with the fingers between the ribs, will generally suffice to indicate the seat of the disease. We refer to the work itself for some cases of phthisis, in which moxa was employed. Four it would appear were cured, and three died of abdominal disease, having nearly recovered from the pulmonic affection.

*Of Chronic and Organic Disease of the Abdominal Viscera.* In cases of thickened pylorus, obstructions of the liver, spleen, "*ou de tout autre viscère de la cavité abdominale*," great success, it is said, has resulted from the use of this favourite remedy, particularly if the disease has not arrived at the last stage of development.

Of Rickets. Both ancient and modern authors have strongly recommended moxa in rickets. Desault remarks, that the remedy is more certainly successful, if the burned parts are not allowed to suppurate. The ammonia is consequently to be applied, in such cases, immediately after the moxa is consumed.

The repetition of the use of moxa will depend upon the obstinacy of the disease. It may be applied during every stage, but of course with more probability of success, during the first period, when the deformity has not proceeded to any great extent. The interval between each application of the moxa must depend upon the age and strength of the patient. It is, of course, more adviseable, that the cure should occupy a longer time, than that the patient should be exposed to the dangers arising from their too rapid application. In " dorsal consumption," to adopt the nomenclature of our author, moxa is " imperiously indicated." This disease, which is better known by the title of curvature of the spine, or the vertebral disease of Pott, has been considered generally incurable. Baron Larrey is of opinion, that abscess invariably results from caries of the vertebræ. We know, however, that such is not invariably the case, we have seen upon dissection caries of the vertebræ without abscess, and we believe that such cases are not uncommon.

Our author observes, that thirty years experience has verified the principles of Pott, respecting spinal disease, and also put him in possession of a sovereign remedy in the repeated application of moxa, which he considers is infinitely preferable to caustic issues. We must pass over the relation of many interesting cases of spinal disease, in which moxa was advantageously employed.

By the term *sacro-coxalgia,* which is the subject of the next section, is implied an affection similar to the vertebral disease occurring in the sacro-iliac symphysis.

Rheumatism is considered a frequent original cause of this complaint, in which, in young subjects particularly, a separation or spontaneous luxation of the bones results. Caries frequently succeeds. A similar luxation has been known to take place in young females, who have given birth to very large children. To establish a diagnosis in this case is considered difficult; local pains increased upon pressure, with a manifest tumefaction of the sacro-iliac region, will indicate its probable existence. Moxa is considered a useful auxiliary in the treatment of sacro-coxalgia.

The sternum and scapulæ are liable to similar attacks of rheumatism, which must be treated in the same manner. "In all these cases, I have observed, says Baron Larrey, that when the abscess, depending upon the caries of the bones, breaks spontaneously, before the caries is arrested by the proper means, that it has terminated fatally, whilst, on the contrary, the early use of moxa, has put a stop to the destruction of the bones, and the abscesses have been opened with success; of this fact I have numerous examples."

*Of Femoro-coxalgia,* or, latent and chronic inflammation of the hip joint," the Baron has witnessed many instances in young soldiers who were much exposed to wet and fatigue. He considers it a rheumatic affection, and observes, that it is characterized by the same symptoms, and requires the same mode of treatment, as those strumous diseases of the hip joint which are so common in children.

The lengthening of the limb which takes place in such cases, is attributed to relaxation and paralysis of the muscles surrounding the joint, and also to a relaxed state of the ligamentum teres. The Baron is not aware of, or at least does not mention, the explanation of this occurrence afforded by John Hunter, which we believe to be accurate; namely, that the *apparent* lengthening of the limb arises from the diseased side of the pelvis becoming lower than the other.

It is the opinion of our author that, "unless from some concomitant mechanical cause, the head of the femur, although previously reduced in its size by caries, is not luxated."—He has never seen a single example of luxation, either in adults or children, although he has had frequent opportunities of opening the bodies of those who have died of this disease. It is, however, an undoubted fact, that dislocation of the head of the thigh bone does take place, in consequence of the destruction of the joint. Mechanical violence may, it is true, be applied, and produce dislocation. A patient labouring under this disease is, however, but little exposed to such a cause. During the first period of the disease, where inflammation of the joint only is to be combated, local bleeding, &c. is recommended. The subsequent use of moxa, the combustion of which is to be kept up by means of the blowpipe, has been highly successful, even when caries and suppuration had taken place.

Baron Larrey cannot yet venture to determine whether in such cases the use of the actual metallic cautery is required: he is inclined to believe "that it may prove a powerful auxiliary to moxa, which, not acting with equal energy, cannot so rapidly arrest the disease." Eight cases are related in which the moxa was successfully applied. Upon white swelling of the knee, we are promised a distinct treatise: it has been cured by moxa. We are now arrived at the conclusion of the Baron's observations upon moxa.

That it is a valuable remedy we do not doubt, and that some of the cases related prove its *occasional* superiority over the other well-known means of exciting counter-irritation is equally certain. In many of them, however, the most powerful and judicious curative measures were resorted to, previous to the application of this highly extolled remedy, and it would therefore be extremely difficult to determine the precise *quantum* of benefit derived from its use.

The high authority of Baron Larrey, will doubtless, be sufficient to induce the surgeons of this country to give it a fair and careful trial. It has hitherto been applied by few English practitioners. The second memoir is "upon the seat and effects of nostalgia, followed by reflections upon partial lesions of the brain, whether resulting from mechanical or spontaneous causes."

Upon the subject of nostalgia, which has been productive of so many conflicting opinions, the author confines himself to the relation of facts; to the careful description of those phenomena which characterize this singular affection, and to the differences which exist between this and other diseases, which also have their seat in the brain. In nostalgic patients the mental faculties first become deranged. The most enchanting and joyous impressions of the beauties of their native place, take possession of their deluded imaginations, however barren it may really be. They conceive their friends and relations to approach them in the richest habiliments, and with the tenderest marks of affection.

This state of excitement is characterized by increased heat of head—rapid pulse—convulsive motions—redness of the conjunctivæ—wandering looks, and precipitate utterance. A state of oppression succeeds, with sighing—costiveness, and wandering pains in different parts of the body.

In the third period there is general debility. The patient is overwhelmed with sorrow; he moans, sheds tears, and loaths his food, and not unusually has a horror of transparent liquids, which gives him an hydrophobic appearance. Life becomes burdensome, and suicide completes the gloomy picture, if paralysis has not deprived the patient of power to execute the deed. The vital energy gradually sinks, and he dies perfectly unconscious of his fate.

It may be easily imagined that during the retreat from Moscow, *nostalgia* was very frequent. We should doubt, indeed, whether there was a single individual in the French army, who would not gladly have yielded all claims to military glory, to have escaped the maddening scenes that must constantly have been presented to his view. Comfortless, indeed, must have been the home which did not inspire the most ardent wishes for return, when placed in such a situation of complicated sufferings. Who would not then have been nostalgic?

In the first stage of nostalgia, bleeding, purgatives, cold to the head, &c. are indicated. In the second, or stage of collapse, the application of moxa is recommended, with light tonics, and change of climate. In the third period art can offer but little assistance; the efforts of nature may produce a favorable crisis. During the whole course of the disease, the utmost kindness, should be shewn to the patient on the part of the attendants. Seven interesting cases of nostalgia are related. Upon dissection of a nostalgic patient, inflammation and suppuration of the brain were the principal morbid appearances.

*Upon the Properties of the Iris.* The object of this paper is to examine the nature of the sympathetic relation which exists between the iris and retina, or optic nerve. Our author is under an error, in supposing that, "until the present day, it has been admitted, that the contractility of the iris depends upon the influence of the optic nerve, or retina." The reverse has been long known. The functions of the iris may be deranged, while the retina remains sound, and *vice versa.* The sympathy between these parts is so great, however, that it will rarely happen that the retina is diseased without corresponding signs of derangement in the actions of the iris. That the iris may be diseased without the retina participating, is a circumstance of very common occurrence, and shews that the sympathy existing between them is not reciprocal. That the motions of the iris are in fact essentially independant of, although intimately connected with, the retina, has been taught in London for the last six years. Baron Larrey has observed several facts which prove that the immobility of the iris does not contra-indicate the operation for cataract. He remarks that the actions of the iris remain perfectly natural during tetanus, although the whole muscular system is affected by spasm.

Several cases also have fallen under his care, in which, from mechanical violence, the iris was completely paralysed; vision remaining perfect. At page 236 it is observed that, "in several affections of the viscera, such as chronic inflammation of the heart, pericardium, liver, &c. the opening of the iris gradually contracts itself, until at last the passage of the rays of light is entirely prevented, whilst the visual properties of the retina preserve their integrity, and sight may be restored by the operation for artificial pupil. It is important to determine, whether vision was perfect before the total occlusion of the pupil, for in the case of amaurosis, the operation would be useless."

Upon this passage we must observe that, as far as our observation extends, a gradual obliteration of the pupil results only from inflammation of the iris, and when it occurs in the diseases mentioned by the Baron, it is almost always accompanied by derangement of the retina. Upon this point, then, we differ from our author. We believe that in the cases to which he alludes, which were relieved by operation, the closure of the pupil was an accidental circumstance unconnected with visceral disease.

That such diseases do frequently lead to loss of sight is certain. This lamentable occurrence, however, arises from a real nervous sympathy, not exciting inflammation which is capable of effecting a permanent closure of the pupil, but, on the the contrary, causing a moderate but permanent dilatation, and terminating in amaurosis, or glaucoma. We must refer to the work for some speculative opinions upon the the cause of the motions of the iris.

A short paper succeeds upon "*Wounds of the Intestines.*" It appears that the Baron is not aware of the progress which has been made in the treatment of such accidents by English authors, particularly Mr. Travers.

The book concludes with a few remarks upon fractures of the neck of the femur, and with some reflections upon the formation of callus in fractures in general. We reserve any observations upon this subject until we are favoured with a separate treatise upon it, which the Baron promises.

To be useful to his fellow creatures is the avowed motive of Baron Larrey, for the publication of the present volume, which contains much valuable practical information. We sincerely believe, that no other inducement would more effectually have prompted his exertions. The determined assiduity and professional ardour of the Baron are the more praiseworthy, as to acquire fame cannot be his object. He already stands at the summit of professional reputation. He has in fact reaped the reward which will rarely be withheld from him who possesses talent, and whose conduct is guided by the principle of our great moralist that "to be idle is to be vicious."

P. S. After the foregoing review of Baron Larrey's work had been drawn up by one of the writers for this journal, we received Mr. Dunglison's translation of the whole of that part of the Baron's work which relates to Moxa, preceded by a learned preface, and accompanied with valuable notes by the translator.* We regret that the extent to which our coadjutor has gone in the present analysis of the memoir on moxa, will render it impossible for us to recur to the subject in a review of the translation. We cannot let this opportunity slip, however, without recommending Mr. Dunglison's work to the attention of our brethren, as very creditable to his talents and judgment, and capable of affording them much important and valuable information.—*Ed.*

---

* On the use of the Moxa, as a Therapeutical Agent. By Baron D. J. Larrey, &c. translated from the French, with notes and introduction, &c. By Robley Danglison, Fellow of the Royal College of Surgeons, 8vo. pp. 148, and 76 pages of preface, London, 1822.

